# Perceived Occupational Benefits and Hazards of Soil Health Practices Among Colorado Farmers and Ranchers: A Qualitative Study

**DOI:** 10.3390/ijerph23080982

**Published:** 2026-07-29

**Authors:** Morgan A. Valley, Ryan Sawyer, Megan B. Machmuller, Helen D. Silver, James Hale

**Affiliations:** 1 Department of Environmental and Radiological Health Sciences, Colorado State University, Fort Collins, CO 80523, USA; 2 Department of Soil and Crop Sciences, Colorado State University, Fort Collins, CO 80523, USA; megan.machmuller@colostate.edu; 3 Ground Up Consulting, LLC, Denver, CO 80206, USA; helene.d.silver@gmail.com; 4 Department of Sociology, Colorado State University, Fort Collins, CO 80523, USA; james.hale@colostate.edu

**Keywords:** agricultural health, soil health, regenerative agriculture, farmer wellbeing

## Abstract

**Highlights:**

**Public health relevance—How does this work relate to a public health issue?**

- Agriculture is among the most hazardous industries in the United States. The occupational health implications of soil health practices, an increasingly promoted approach to sustainable farming, have not previously been examined.
- Farmers and ranchers implementing soil health practices described a range of perceived occupational health benefits and hazards spanning physical and psychosocial domains, alongside largely informal safety management practices.

**Public health significance—Why is this work of significance to public health?**

- Participants highlighted psychosocial benefits including meaningful work, connections with nature, and community that may serve as protective factors against the well-documented mental health challenges facing agricultural producers.
- Soil health farmers and ranchers reported limited PPE use due to perceptions of reduced synthetic chemical risk, a finding that warrants further investigation into occupational hazard awareness and safety practices in this population.

**Public health implications—What are the key implications or messages for practitioners, policy makers and/or researchers in public health?**

- Farmers and ranchers implementing soil health practices framed their well-being as interconnected with the health of their soil, animals, and ecosystems, reflecting a One Health orientation that public health researchers and practitioners can engage with in the design of research, outreach, and programming.
- Agricultural health and safety programs should develop guidance tailored to the specific hazard profile of soil health farming, including respiratory exposures from organic materials and the musculoskeletal demands of increased manual labor, rather than relying solely on conventional farming safety frameworks.

**Abstract:**

Soil health practices are increasingly promoted through sustainable agricultural programs to improve environmental outcomes, yet little is known about how these practices impact the occupational health of farmers and ranchers who implement them. This qualitative study examined perceived occupational benefits and hazards of soil health practices among Colorado farmers and ranchers. Semi-structured interviews with 15 Colorado farmers and ranchers were conducted between October 2024 and January 2025 and then analyzed using thematic analysis. Six themes were identified: perceived physical benefits, psychosocial benefits, physical hazards, psychosocial hazards, and health and safety practices, and One Health perspectives of the impacts of soil health. Participants described reduced chemical exposure, increased physical activity, and improved mental health, creativity, connection to nature, and community as perceived benefits. Perceived physical hazards included heat stress, respiratory exposure to mold from organic mulching materials, and musculoskeletal strain. Perceived psychosocial hazards included social isolation, financial uncertainty, and a sense of insufficient collective action. Health and safety practices used were largely informal, and personal protective equipment use was limited. Participants described their health as intertwined with the health of the land and living systems around them, reflecting a One Health orientation. Findings suggest that soil health practices may alter occupational health risk in ways that warrant further investigation and tailored safety guidance and programmatic support.

## 1. Introduction

Observations on the connections between soil and human health date back to antiquity, and numerous studies have examined the environmental impacts of agricultural practices that can improve soil health and crop production [1,2,3]. A growing body of evidence points to the soil microbiome as a particularly important mechanism linking soil and human health. Soil hosts diverse microbial communities that influence human health through their effects on the productivity of crops, their role as a source of compounds for treating human disease, and their relationship to the human gut microbiome and immune function [4,5]. These connections underscore the relevance of a One Health perspective, an integrated framework recognizing that the health of humans, animals, and ecosystems are interdependent and must be addressed together [6]. Despite this growing evidence base, the current literature on how agricultural practices that promote soil health affect human health focuses broadly on populations and ecosystems and does not include much evidence on the experiences of the agricultural producers who enact the practices.

Efforts to improve soil health have been widely promoted as part of sustainable agricultural programs at local, state, national, and international levels. For example, in Colorado, the Colorado Soil Health Program [7], run through the state department of agriculture, provided financial and technical assistance to over 400 producers since 2021 to implement new soil health practices and to encourage the long-term adoption of these practices across their operations. Core soil health practices include keeping the soil covered with either mulch or other crops to maintain crop residues, minimizing soil disturbance by reducing tillage, increasing plant and livestock diversity, using cover crops and maintaining live crop roots in the soil year-round, which promote improved nutrient cycling and water retention, while reducing erosion and suppressing weeds [8]. The implications of these programs for farmer health and safety remain underexplored.

Agriculture is among the most hazardous industries in the United States, with some of the highest rates of occupational injury and illness across sectors [9]. Injuries and illnesses in agriculture result from a range of exposures including machinery accidents, falls, excessive heat, repetitive motion, and pesticide exposure [10]. Beyond physical hazards, farming is also associated with significant psychosocial burdens. Farmers globally face elevated rates of depression, anxiety, burnout, and suicide compared to the general population, driven by financial volatility, climate uncertainty, social isolation, and the identity-defining nature of agricultural work [11,12]. Understanding how specific farming approaches, including those that prioritize soil health, shape these occupational health risks and benefits is therefore an important public health issue.

To date, only a handful of studies have addressed the implications of agricultural practices that prioritize soil health on the health of people who work in agriculture. A search of the peer-reviewed literature identified a small number of studies, summarized below, that have examined occupational or well-being outcomes specifically in relation to soil health-promoting practices, in contrast to the larger studies on organic farming and on agricultural occupational health more broadly. For example, Brown and colleagues [13,14] have studied how sustainable agricultural practices, including those that prioritize soil health, may support aspects of farmer well-being through increased self-efficacy, which is a farmer’s sense of their ability to successfully manage land for a range of outcomes and adapt to changes. Similarly, farmers who prioritize and are satisfied with their soil conservation practices report increased levels of job satisfaction [15], which has been associated with overall quality of life satisfaction [16].

The occupational health and safety impacts of organic farming practices, which overlap with soil health practices by minimizing chemical inputs and fostering biodiversity, have been investigated. Given the absence of a substantial body of occupational health research specific to soil health practices, the organic farming literature offers a relevant evidence base for soil health-promoting farming, although a farmer can adopt one without the other. A recent systematic review by David et al. [17] compared the health of farmers in organic versus conventional farming systems. The review found that organic farmers had better psychological and physical health compared to conventional farmers. However, one study included in the review [18] found that conventional farmers reported significantly more pesticide-related symptoms (skin rashes, headaches, dizziness), while organic farmers experienced more musculoskeletal complaints in multiple body regions, suggesting different occupational health risk profiles between the two farming practices. Moreover, organic farmers have reported high rates of non-fatal workplace injuries and limited use of safety practices [19]. These mixed results, and the limited focus on soil health practices specifically, warrant further investigation. Much of the existing research on farmer health relies on quantitative and epidemiological approaches [11], leaving the lived experiences and perceptions of farmers, including their understanding of the hazards and benefits of their own practices, underexplored. A qualitative approach is well suited to capturing these perspectives and can inform outreach and programmatic support to further enhance protections for farmers who implement soil health practices.

This qualitative study investigates how farmers and farm managers in Colorado perceive the occupational benefits and risks associated with implementing soil health-promoting practices.

## 2. Materials and Methods

The research team included faculty, staff, and an undergraduate student from two centers: one that researches and promotes agricultural health and safety; and the other that researches and promotes soil health practices in Colorado.

Participants were recruited through the existing networks of the study team and state organizations, such as the Colorado Department of Agriculture’s Soil Health program. Participants who were interested in participating completed a screening survey and then were contacted by the research team to schedule interviews. A total of 41 individuals completed the screening survey; 26 were excluded prior to interview (14 were identified as likely bot or fraudulent survey submissions, 6 reported farming or ranching outside Colorado, 4 were unavailable during the scheduling window, and 2 had not yet begun farming), resulting in a final sample of 15 participants. Inclusion criteria for the interviews for participants were: (1) aged 18 years of age or older, (2) self-report as a farmer, rancher, or farm manager who uses practices to sustain or improve soil health, (3) work in Colorado, and (4) speak English. This study examined perceptions of health and safety impacts of the soil health practices that participants reported currently implementing on their own operations (reported in Table 1), rather than all soil health practices described in the broader literature.

Semi-structured interviews were conducted by phone or through Microsoft Teams between October 2024 and January 2025 and took 30–50 min. Before each interview, the research team obtained informed consent from participants and confirmed their agreement to be recorded. Participants received a $50 gift card. Interviews were recorded, and interview transcripts were de-identified. The Colorado State University Institutional Review Board reviewed the study protocol (#6100) and approved it as exempt.

The interview guide included open-ended questions asking participants to describe their perceptions of the connections between soil health and farmer health with specific questions about their physical and psychosocial health impacts. Additionally, participants were asked about their safety practices and use of personal protective equipment. The interview guide allowed interviewers to probe on topics that participants raised. The guide covered three areas with open-ended questions: (1) transition to soil health practices, including motivations and perceived changes to self, farm, and community; (2) farmer health and safety, including perceived connections between soil and human health, physical and mental health changes, dust and respiratory exposure, and PPE use; and (3) a closing section on the meaning of farming work and broader societal conditions. Interviewers probed to keep responses anchored to soil health practices. The interview guide and screening survey are provided as Supplementary File S1.

Transcripts were analyzed using deductive followed by inductive thematic analysis [20]. The initial codebook was based on the interview guide and perceived health hazards and benefits. Transcripts were coded by two research team members using Dedoose qualitative software Version 10.0.59 [21]. The team used an iterative process of coding transcripts independently and discussing coded transcripts to reach agreement and to refine the codebook. Disagreements in coding between the two team members were resolved through discussion until consensus was reached. All 15 interviews were included in the final thematic analysis. After all transcripts were coded, coded excerpts were reviewed to identify themes. Consistent with conventions for thematic analysis of semi-structured interview data [22], themes were developed based on patterns of meaning across the dataset rather than on the frequency with which a topic was raised by individual participants. Because interviewers probed flexibly based on what participants raised, not every participant was asked about every topic in equal depth, so the absence of a comment on a given theme does not necessarily indicate the absence of the underlying experience.

## 3. Results

### 3.1. Participant Characteristics

Participants included 15 farmers and ranchers based in Colorado (Table 1). Participants’ ages ranged from 30 to over 70, with a third (*n* = 5) between the ages of 30 and 39. More than half the participants (*n* = 8) had been farming or ranching for 10 or more years, with three participants reporting 30 or more years of experience. Participants produced a diverse range of commodities (vegetables (*n* = 10) and livestock products (*n* = 9)) on a range of sizes of operations (plots less than 50 acres (*n* = 8) to more than 1000 acres (*n* = 3)). Nearly all participants reported using the core soil health practices of minimizing soil disturbance (*n* = 14), increasing plant or animal diversity (*n* = 14), using soil cover (*n* = 12), or cover crops (*n* = 13). Seven participants reported additional soil health practices beyond these four core categories. These practices included soil amendment and use of biological inputs, including compost (in solid and tea/extract forms), chicken litter and cow manure, mushroom substrate, and mycelium or other microbial inoculants intended to improve soil chemistry, structure, and biology, as well as intercropping. Fourteen of the 15 participants reported currently testing their soil to inform their practices.

### 3.2. Thematic Findings

Thematic analysis of interview transcripts revealed six primary themes related to the occupational health experiences of farmers and ranchers implementing soil health practices: perceived physical benefits, perceived psychosocial benefits, perceived physical hazards, perceived psychosocial hazards, and health and safety practices. A subset of participants reported perceiving few or no occupational hazards associated with their soil health practices. Across themes, participants situated their own health within a broader ecological context, describing connections between soil health, food quality, animal health, and human well-being that reflect a One Health perspective. All 15 interviews contributed to the analysis, and each theme described below reflects a pattern raised across multiple interviews rather than the account of a single participant. Each theme is described below with representative quotes from participants.

#### 3.2.1. Perceived Physical Benefits

Participants described three areas that they perceived as the physical benefits of soil health practice. Specifically, they described reduced exposure to chemicals because of minimal use of synthetic fertilizers and pesticides, increased physical activity, and consuming nutritious food grown on their own operations.

Participants perceived that soil health practices reduced their exposure to synthetic chemicals and pesticides. For example, one participant said, “On my health and safety well, [the biggest benefit is] probably not using synthetic fertilizers. Not being exposed to that. I’m not certified organic, but I try to follow those practices. I would assume that that has a positive impact on my health, just not dealing with the toxicity of certain products.” Another participant explained how healthy soil reduced their need for chemical interventions, which they believed benefits the farmer and the food supply, saying, “To have a healthy soil, it needs to be minerally balanced, biologically alive, mechanically open so it’s not compacted. I’m looking at it as a multifaceted issue. […] We can get away with really using minimal pesticide interventions or plant disease interventions. And so, if my plants are healthy, my farming is healthier for me and for everyone around me and the food that people get to eat is better for them because it’s of a higher quality.”

Participants also described soil health practices as contributing to higher levels of physical activity during their workday. One participant reflected on the additional exercise in their daily routine, stating, “Well, I stay pretty active according to my phone. I walk an average of eight to ten miles a day. I don’t actually have a workout or exercise program, but I’m very active in my job and outside and moving a good portion of the day, so I think that contributes to my health.” Another participant stated that soil health practices encourage physical activity, saying, “I think a lot of [soil health practices] require getting out of a machine. Moving our bodies around is good, right? Functional movement, I think they call it. I’m really grateful that the type of farming I do has me moving around.”

Several participants described consuming food grown on their own operations as a perceived health benefit, with some connecting soil health directly to the perceived nutritional quality of the food they eat. As one participant said, “We eat food from the farm, and I assume that food is healthier and potentially more nutrient dense than you know food grown on soil that’s really beat up.” Another participant described a perceived link between soil health and human nutrition, saying, “I think there’s a huge connection between the minerals and the nutrients that are in the soil and the minerals and nutrients or depletions that we see in human health and in our food.”

#### 3.2.2. Perceived Psychosocial Benefits

In addition to physical benefits, participants described a range of psychosocial benefits associated with soil health practices, including positive effects on mental health and creativity, connections with nature, meaningful work, and a sense of community.

Some participants described soil health practices as contributing to their psychological well-being and fostering a sense of creativity in their work. As one participant said, “Doing it this way, for the health of the soil naturally, is absolutely healing to one’s psyche and soul.” Another participant described how the creativity required by soil health practices extended into farm management more broadly, saying, “I think in the same way that focusing on soil health or regenerative practices represents a departure from the status quo and being creative and flexible, I think you can extend that more broadly to marketing or whatever the task is. It’s not just necessarily production focused, but how you sell what you’re producing and think about the industry landscape. All these things fit together, and I think can certainly mitigate some of that powerlessness that people oftentimes feel that results in mental health crises.”

Participants also described a sense of connection with nature as a dimension of their well-being as soil health farmers. One participant described the restorative quality of working outdoors, saying, “I’m out in nature. I can see growth, life, and those sorts of things. So I think that brings a peace and comfort which would, I think, help mental health. We tend to be profitable and aren’t stressed by financial constraints and that sort of thing. So overall, I think just being in this environment is very soothing, comforting, and contributes to good mental health and physical health.” Another participant described feeling a sense of well-being due to their reciprocal relationship with the land. They said, “Working with the land gives me the ability to be outside and commune with nature. You can literally tell that when you’re making improvements to the land, the land responds in a positive way, and that’s really cool. It’s almost like a communication with the land. So yeah, that part really means a lot to me. When you do something good for the land, it responds, and it tells you that it likes it and that it’s working overall better.” Another participant described their connection to the land in terms of generational ties, saying, “When I go irrigate out in the summertime, I stick my hands in the mud where the water is at that I just got done setting an irrigation tube, and I feel that dirt running through my hands. That is connection to my father, to my grandfather, to my great grandfather that were all farmers in Colorado going back to the early 1900s. That is the connection that I have when I can feel that wet soil in my hands, that is the connection. I want my nephews to have that same connection when they run their fingers through that soil, whether it’s wet soil, whether it’s dry soil, I want them to have that same connection and knowing that this farm goes back generations and that they can keep this farm going for the next future generations, that’s the connection.”

Participants described a sense of meaningful work as another psychosocial benefit of soil health farming. One participant described farming in a way that promotes soil health as a particularly fulfilling occupation, saying, “Farming is just one of the most, I think, fulfilling occupations you can have, and to me that really helps with my own mental and physical health. Being outside every day, using my body, being close to nature, I think those are all important qualities in overall health.” Another participant described the meaning they derived from caring for the land and their community, stating, “I would say it’s very meaningful, not only to me but hopefully to the community around me. I’m part of a ranch that wants to have healthy ground and healthy animals for people to consume, and we’re trying our best to do right by the land.”

Participants also described soil health practices as fostering meaningful connections with others and a sense of community. As one participant said, “I get to have a big impact on community. We get together, we have the joy of growing food together, and we care for the environment together and also celebrate together. We get to build meaningful relationships by doing very meaningful work. So I think working with the land to me is just such a nice way of being in the world.”

#### 3.2.3. Perceived Physical Hazards

Participants described several perceived physical hazards associated with soil health practices, including heat stress, respiratory exposures from mulching materials and soil amendments, and musculoskeletal strain and increased labor demands.

Some participants identified heat as an occupational concern that is shared with farmers and ranchers more broadly, particularly in the context of a changing climate. One participant described the adaptations they anticipated making to protect workers, saying, “I think if Colorado gets hotter and hotter, that’s another thing we’re going to have to be mindful of by starting earlier, working, taking a break, working later. Which is really hard for my form of farming. So it’s really just trying to protect my staff from heat exhaustion.”

Respiratory exposures were also raised as a perceived concern. One participant described inhaling mold and mildew from organic mulching materials as a potential occupational hazard. They said, “I do breathe in some of the mold and mildew that will be on some straw that I’ve used to mulch with. We will get organic straw reduced in price to their bottom bales, and so they have some moisture and are a little moldy or mildewy. We do breathe that in a little bit. I’ve gone to sourcing less moldy, less prone to mold, straw for that reason.” Similarly, another participant said, “If you’re using certain things like brewing compost or fermenting microbial amendments, teas, and stuff, there’s a chance of creating disease-causing organisms.”

Musculoskeletal strain was identified as an enduring physical hazard of farming. As one participant said, “Farming is physically hard on the body. I think regenerative practices help, but they don’t remove the physical strain that farming puts on you.” Another participant noted that the commitment to soil health practices can increase the labor intensity of farm work, stating, “Valuing soil health and implementing practices that preserve soil is a lot more labor intensive [than conventional farming]. And so there are some times where you’re like, ‘Oh, I’m making things a lot harder on myself to farm regeneratively.’”

#### 3.2.4. Perceived Psychosocial Hazards

Beyond physical hazards, participants described psychosocial challenges associated with farming with a soil health focus. These included social isolation and economic precarity, financial stress and uncertainty related to adopting new practices, and a sense of insufficient collective action from consumers, markets, and policymakers.

Some participants described the emotional toll of work that can feel isolating and economically precarious. As one participant said, “It’s really isolating, lonely work sometimes, and it’s thankless, especially when trying to make a living in a global economy where you can almost never compete for a livable wage.” Others described feeling frustrated that their individual efforts toward soil health might be insufficient without broader systemic change. One participant stated, “I feel like I’m trying to do my job in a way that will help humankind, but it doesn’t feel like enough unless we’re all in it together. We need consumers to choose products supporting soil health practices and government incentives to help farmers switch to more sustainable practices.” Another participant described the stress of market pressures and the misuse of soil health terminology, saying, “The pressures from the economy are what are stressful. So, for instance, it’s stressful when some business goes out there and slaps regenerative on something that’s not actually regeneratively grown. Or they think regenerative is a synonym with organic. It’s stressful when people just really want the cheapest food, even when they’re affluent. That’s what troubles me most is when the affluent members of our community are complaining about food prices.”

Financial risk and uncertainty emerged as a significant psychosocial stressor as well. One participant described the fear associated with transitioning away from conventional practices, saying, “Changing practices is terrifying for farmers. The uncertainty of the financial risk is a huge barrier for many.”

#### 3.2.5. Limited or No Hazards

Not all participants identified occupational hazards associated with soil health practices. Several described their operations as presenting few or no meaningful health or safety risks, often attributing this to the small scale of their farms or the nature of their soil health approach. One participant said, “I don’t think I have any [health or safety concerns] because we’re so small. We don’t use equipment where I could get run over by it.” Another participant expressed a similar view, stating, “Ultimately, I don’t see health or safety risks [to soil health practices].” A third participant cited no health risks other than the workload, saying, “I think there are health benefits [to soil health practices]. I don’t see any risks health-wise other than maybe it might be more work [than conventional farming practices].”

#### 3.2.6. Health & Safety Practices

Participants described a variety of health and safety practices used on their operations. Many were framed as common sense measures based on experience rather than formal training and reflected general farming safety rather than practices specific to soil health farming. Participants also described limited use of personal protective equipment, which they linked directly to the reduced chemical inputs associated with soil health practices specifically.

General farming safety practices reported by participants primarily focused on equipment awareness and clear communication. As one participant said, “You could roll over on a tractor. I lecture people about machine safety. I say, ‘When I’m on that tractor, I can’t see everything. It’s your job on the ground to stay away from me.’” Another participant framed safety practices more broadly as a matter of basic awareness around farm equipment, stating, “Well, with safety a lot of it just goes back to common sense. I mean, you don’t want to stick your hand in an auger. You don’t want to stick your hand in a belt.” Another participant described safety communication across multiple hazard areas, including equipment and livestock, stating, “We try to advocate and talk about safety and have some instructions for safety regarding operating equipment such as tractors, ATVs, trucks, working around livestock and that sort of thing.”

Heat and physical strain were addressed through a mix of informal and formal approaches. One participant described relying on worker self-reporting and task flexibility, saying, “We’re just asking people to be very honest about how they’re feeling, take a lot of water breaks to step out of the field into the shade. If their body hurts, if they need to do a different position, [I ask employees] to come and find me so I can maybe give them a different job if they want to continue.” Two participants described more formalized training programs, with one outlining multiple required trainings covering heat illness and food safety, stating, “We go through heat safety training that’s mandated by the state of Colorado to ensure everybody understands what they need to do to prevent heat illness. We also do a food safety training because in the wash station we use some caustic sanitizers. And also, I was just learning more about foodborne illness and health and hygiene, because I want to have a safe food product.”

Participants described limited use of personal protective equipment (PPE), with most perceiving PPE as unnecessary given the few chemical inputs associated with soil health practices. As one participant said, “I feel like that’s [PPE] not really applicable to our kind of ranching. We’re not spraying or using equipment much.” Another participant shared a similar approach, noting certain exceptions, stating, “If I was going to be in a very dusty situation, I’d probably wear a dust mask or something like that. Since we use very few chemicals on the land, we don’t feel like there’s a need to wear protective clothing or masks like you would if you were spraying herbicides or pesticides.”

In contrast, one participant described a more comprehensive approach to PPE in the context of extreme weather, saying, “We just went through some subzero weather this past week. We tried to ensure that everybody has adequate water. When it’s severe weather circumstances such as extremely high winds or extreme cold, we ensure that everybody’s wearing protective cover and try to stay out of it as much as possible. We usually don’t send anyone out alone in conditions like that where they may become isolated or abandoned.”

#### 3.2.7. One Health Perspective of Soil Health Impacts

Participants described the health impacts of soil health practices in relation to the health of animals, ecosystems, and broader communities, reflecting a One Health perspective and a sense of their work as stewardship within natural systems. While this theme shared some conceptual ground with the physical and psychosocial benefit themes described above, it distinctly reflected how participants situated their personal health within a broader web of animal, ecosystem, and community health, often without reference to a specific personal benefit or hazard. For example, one participant described how more intensive land and livestock management created a feedback loop of improved animal health and reduced farmer stress. They said, “Generally, through more intensive management, the need for more intensive observation and record keeping is positive because you’re out there more. Managing cattle more intensively, being around them more, ensuring it’s not just about moving them frequently but keeping better tabs has better health outcomes for them, which reduces our stress. There’s a degree of pleasure you can get from participating in a thriving ecosystem, feeling like an active participant in stewardship rather than just an observer. That has substantial mental health benefits.” Another participant described their entry into agriculture as motivated by a wanting to connect soil, animal, and human health, saying, “I got into agriculture because I saw people grazing animals in a way that hopes to create a holistic approach, moving animals on the ground to help plants, water, and minerals. As a young person, I felt like this was a way I could help the planet, soil, animal health, and human health.”

Several participants also described their relationship to the land and broader ecosystem as one of humility and stewardship rather than dominance or control. As one participant said, “I think also having the humility to recognize that we as humans are pretty limited in our ability to fully grasp the complexity of natural systems. That’s important to me too, the humility aspect and just seeing myself as something else out there, similar to the cattle or the birds or fungi, whatever. Just out there making decisions and doing things that hopefully have a net positive benefit on the whole.” Another participant described their role as an active participant in a larger ecological system, stating, “The concept of stewardship is really important, and I really don’t see that as being a kind of dominant relationship with the land or the broader ecology. I guess what’s most meaningful to me is thinking about it [farming] as just being an active participant in a much broader and more complex system and that I have the ability to influence it in pretty profound ways, whether I’m aware of it or not.”

Participants also described the interconnection between soil health, food, and community health. One participant described how prioritizing soil health benefited the health of the people and community they serve, saying, “I feel like the people that understand the importance of soil health also understand how interconnected things are, and that goes along with mental and physical health, I think. If you’re providing more nutritious food for the community, the community supporting you producing healthy and nutritious food. I think this area [in Colorado] is especially special in that way, just because the people here are willing to support farms that are doing great work in maintaining soil health.” Another participant described a broader view of the relationship between humans and the land, stating, “I think human beings are much more connected to the land than we ever imagined. And culturally, we’re getting much more divorced from the land. I think that the benefits to our mental health and souls from doing important, meaningful work, ideally helping the environment and at the least, not damaging it.”

## 4. Discussion

This qualitative study examined how farmers and ranchers in Colorado perceive the occupational health benefits and risks associated with implementing soil health practices. Thematic analysis revealed six primary themes: perceived physical benefits, perceived psychosocial benefits, perceived physical hazards, perceived psychosocial hazards, and health and safety practices, as well as an overarching One Health perspective that framed participants’ understanding of the connections between soil, animal, and human health. These findings add to the limited but growing body of literature on the occupational health implications of sustainable agricultural practices and have implications for research and outreach programming.

### 4.1. Perceived Occupational Benefits

Participants consistently identified reduced exposure to synthetic chemicals and pesticides as a primary perceived physical benefit of implementing soil health practices. This perception aligns with existing evidence that organic farmers, whose practices can overlap with soil health approaches, report fewer pesticide-related symptoms than conventional farmers [17,18]. Participants described reduced chemical use not only as a personal health benefit but as one that extended to their workers, surrounding communities, and the quality of the food they produce, a framing consistent with a One Health perspective in which the health of the individual farmer, the food system, and the environment are interconnected [4,5]. Because organic certification and soil health practices overlap in their reduction in synthetic chemical inputs, evidence from the organic farming literature is applicable to the chemical-exposure-related benefits described here. However, this applicability is more limited for other dimensions of soil health practice, such as reduced tillage, cover cropping, which are not required for organic certification and are not consistently captured in organic-versus-conventional comparisons. Findings drawn from the organic farming literature should therefore be interpreted as suggestive rather than directly generalizable to the full range of soil health practices examined in this study.

Participants also described soil health practices as contributing to higher levels of physical activity through hands-on, equipment-light farming approaches. Several described consuming food grown on their own operations as a perceived health benefit. While these are perceived rather than clinically verified benefits, they reflect participants’ understanding of soil health as directly connected to their own physical well-being.

In addition to physical health benefits, participants described a range of psychosocial benefits including improved mental health, creativity, connections with nature, meaningful work, and a sense of community. These findings are consistent with existing literature suggesting that sustainable agricultural practices support farmer well-being through increased self-efficacy and job satisfaction [13,14,15,16]. The present findings extend this evidence base by suggesting that soil health practices foster creativity and social connection, psychosocial resources that are particularly significant given the elevated rates of depression, anxiety, and suicide documented among agricultural producers [11,12]. Whether soil health practices represent a modifiable protective factor for farmer mental health warrants further investigation.

Participants’ descriptions of their connection with nature as a psychosocial benefit of soil health farming are consistent with evidence documenting the mental health benefits of nature contact [23]. Research suggests that individuals who are more connected to nature derive greater psychological well-being from nature exposure, particularly when that engagement is intentional rather than incidental [24]. Farmers and ranchers who implement soil health practices may be particularly well positioned to experience these benefits, given the hands-on, observational nature of their work and the close attention to living systems that soil health practices require. The generational dimension of nature connection described by some participants, in which contact with the soil evoked a sense of continuity with family members who had farmed the same land, suggests that for some producers this connection extends beyond individual well-being to aspects of identity and legacy, which has been associated with both positive and negative mental health outcomes [25].

Participants’ descriptions of farming as deeply meaningful work represent a distinct psychosocial benefit, consistent with evidence that perceived meaning and purpose in work are longitudinally associated with better mental well-being [26]. Several participants framed their work in terms that align with the construct of calling, a sense of being drawn toward purposeful, prosocial work [27]. The emphasis participants placed on caring for the land and producing nutritious food for their communities reflects the prosocial, purpose-driven dimensions of meaningful work that have been linked to well-being [28]. That participants often described this sense of meaning as inseparable from their connection to nature suggests the two may operate as mutually reinforcing psychosocial resources rather than as independent benefits.

### 4.2. Perceived Occupational Hazards

Despite these perceived benefits, participants identified several potential physical occupational hazards associated with soil health practices including heat stress, which is a well-documented agricultural risk factor [29], and respiratory exposure to mold and mildew from organic mulching materials. Mold and mildew exposure from mulching materials was raised by a subset of participants. This finding is consistent with the established literature on organic dust toxic syndrome (ODTS), a documented occupational illness among agricultural workers caused by inhalation of mold spores and organic dust from materials including hay, straw, and mulch [30,31]. Notably, a cluster of ODTS cases among landscape workers in Colorado was previously linked to contaminated mulch containing high levels of Aspergillus spores and endotoxin [30]. The present findings extend this concern to farmers using organic mulch as a soil cover practice, suggesting that this common soil health strategy may introduce a respiratory hazard that warrants attention in agricultural health and safety guidance specific to soil health farming systems.

Musculoskeletal strain was identified as an enduring agricultural hazard that soil health practices do not eliminate and in some cases intensify through increased manual labor demands. This finding is consistent with evidence that organic farmers report greater musculoskeletal complaints and more frequent exposure to vibration than conventional farmers [17].

Participants also described psychosocial challenges including social isolation and financial uncertainty around transitioning to new practices, consistent with the established literature on farmer mental health [11,12]. Notably, some participants noted a tension between their personal commitment to soil health and the perceived absence of collective systemic support from consumers, markets, and policymakers. Taken together, these findings suggest that soil health practices may shift the occupational risk profile of farm work in ways that deserve targeted attention from agricultural health and safety programs, although the psychosocial burdens reflect the structural conditions in which soil health farmers operate and cannot be addressed by individual-level interventions alone.

### 4.3. Health and Safety Practices

Many of the participants’ descriptions of health and safety practices reflected an informal, experience-based approach, consistent with the broader agricultural occupational health literature documenting limited adoption of formal safety practices in farming communities [19]. While a few participants described formal training programs, including state-mandated heat illness and food safety training, others relied primarily on common sense and accumulated experience. Personal protective equipment use was similarly limited, with participants perceiving PPE as unnecessary given the reduced chemical inputs associated with soil health practices. Formal guidance specific to the hazards associated with soil health practices, including respiratory exposure from organic mulching materials, musculoskeletal demands of increased manual labor, and appropriate use of PPE, was not described by participants, suggesting this may be an area where agricultural health and safety resources have not yet reached this population.

These findings have several practical implications for agricultural health and safety programming. First, existing extension and outreach materials on PPE and chemical safety are largely framed around pesticide and synthetic input use; these materials may be perceived as irrelevant by soil health farmers who use few chemical inputs, even though they may face other hazards, such as respiratory exposure to organic dust and mold from mulching materials and amendments, that current guidance does not address. Agricultural health and safety programs serving this population may need to develop or adapt materials that speak specifically to the hazard profile of soil health practices rather than relying on conventional, pesticide-focused safety messaging. Second, the informal, experience-based safety culture described by participants suggests an opportunity for programs such as Cooperative Extension, state soil health initiatives, and regional agricultural safety and health centers to integrate basic safety guidance into existing soil health technical assistance and outreach, reaching producers through channels they already use rather than requiring separate safety-specific engagement. Third, given that several participants described psychosocial benefits such as meaning, creativity, and connection to community alongside psychosocial hazards such as financial uncertainty and isolation, programs supporting the adoption of soil health practices may benefit from pairing technical assistance with attention to the financial and social conditions that shape farmer well-being, rather than treating physical safety and behavioral health as separate domains.

### 4.4. One Health Perspective

An important finding of this study was the One Health perspective that participants expressed across interviews. Participants consistently described their own health as interconnected with the health of their soil, animals, and the broader ecosystem. This alignment between farmers’ experience and the One Health framework, which formally recognizes the interdependence of human, animal, and environmental health [6], has implications for how agricultural health research and programming are framed. The National Academies of Sciences, Engineering, and Medicine [4] have called for greater integration of soil health into One Health research and practice, and the present findings suggest that farmers who implement soil health practices are already operating from an intuitive One Health orientation that can inform the design of research and outreach efforts.

### 4.5. Limitations and Strengths

This study has several limitations that should be considered when interpreting its results. The sample was small and purposive, recruited through existing networks and the Colorado Soil Health Program, which introduced the potential for self-selection bias toward participants with positive views of soil health practices. The findings reflect the perceptions of a specific group of Colorado producers and may not transfer to farmers in other geographic or agricultural contexts or among farmers with different demographic characteristics. As a qualitative study, the findings describe perceived rather than objectively measured health outcomes, and the connections participants drew between soil health practices and their health reflect their own interpretations rather than causal relationships. Although interviewers anchored questions in soil health and redirected participants who broadened into general farming experiences, some participant responses likely reflect occupational benefits and hazards of farming more broadly rather than soil health practices specifically. This overlap is difficult to fully disentangle in semi-structured interviews and should be considered when interpreting findings that closely resemble general agricultural health literature.

This study also has several strengths. The use of semi-structured interviews allowed participants to raise topics and concerns in their own words, producing rich, contextualized data that survey-based approaches would not have captured. The interdisciplinary composition of the research team, which included expertise in both agricultural health and safety and soil health, strengthened the relevance and credibility of the interview guide. Together, these features support the trustworthiness of the findings as an initial qualitative account of a topic that has received little empirical attention.

### 4.6. Future Research Directions

Future research building on these findings could take several directions. Quantitative and mixed-methods studies could examine associations between specific soil health practices and measured occupational health outcomes, including injury rates, respiratory health, and musculoskeletal disorders. Research with larger and more diverse samples, including farmworkers and producers in other regions, would broaden and strengthen the evidence base.

The respiratory hazard associated with organic mulching materials warrants dedicated investigation in the specific context of farming that intentionally promotes soil health. While ODTS from organic dust exposure is documented in agricultural settings [30,31], the prevalence and severity of respiratory and biological hazard exposure among farmers using mulch and fermented amendments as soil health practices has not been examined. Studies assessing exposure levels, symptom prevalence, and effective mitigation strategies in this population would fill an important gap and inform safety guidance tailored to soil health farming operations.

Longitudinal research examining how adoption of soil health practices changes occupational health and wellbeing over time would be particularly valuable, given that practice transition is a period of potentially heightened stress and uncertainty. Finally, future research should explore the extent to which farmers’ intuitive One Health orientation influences their health behaviors, safety practices, and engagement with agricultural health programming.

## 5. Conclusions

This qualitative study examined how farmers and ranchers in Colorado perceive the occupational health benefits and risks of implementing soil health practices. Participants described a range of perceived benefits and hazards spanning physical and psychosocial domains, alongside largely informal approaches to health and safety management.

Participants described soil health practices as a source of meaning, creativity, connections with nature, and community, factors that may serve as protective resources against the well-documented mental health challenges facing agricultural producers. At the same time, financial uncertainty, social isolation, and a sense of insufficient systemic support represent psychosocial hazards that programs supporting soil health adoption should seek to address. Low PPE use among participants who perceived few or no occupational hazards highlights the need for safety education tailored to the specific risk profile of soil health farming.

Across themes, participants described their health as connected with the health of the soil, plants, and animals on the farm, reflecting an intuitive One Health orientation. Future research with larger and more diverse samples, including quantitative and longitudinal approaches, is needed to establish a more robust evidence base for protecting and promoting the health of farmers and ranchers who prioritize soil health. Based on participants’ accounts of unaddressed hazards and the informal channels through which they currently receive information and support, tailored safety guidance for soil health practices, integration of safety messaging into existing technical assistance and attention to the financial and social conditions affecting farmer well-being could benefit this population.

## Figures and Tables

**Table 1:** Characteristics of participants.

Age	Number of Interviewees (*n* = 15)
<30 years	0 (0%)
30–39 years	5 (33%)
40–49 years	3 (20%)
50–59 years	3 (20%)
60 years or older	4 (27%)
**Years in Farming**	
<1 year	0 (0%)
1–4 years	1 (7%)
5–9 years	6 (40%)
10–19 years	4 (27%)
20–29 years	1 (7%)
30+ years	3 (20%)
**Acreage**	
<50 acres	8 (53%)
50–499 acres	3 (20%)
500–999 acres	1 (7%)
1000 or more acres	3 (20%)
**Land Ownership**	
Own Land	4 (27%)
Lease Land	3 (20%)
Manage Land	4 (27%)
Both Own and Lease Land	4 (27%)
**Commodities Produced**	
Grains and Feeds	5 (33%)
Livestock Products	9 (60%)
Fruits	5 (33%)
Vegetables	10 (67%)
Legumes	3 (20%)
Nuts, Forestry, Fiber, & Fuel Products	4 (27%)
Other	2 (13%)
**Soil Health Practices Used**	
Minimize Soil Disturbance	14 (93%)
Increase Plant/Animal Diversity	14 (93%)
Incorporate Cover Crops	13 (87%)
Increase Soil Cover	12 (80%)
Other	7 (47%)
**Currently Test Soil**	14 (93%)

## Data Availability

Upon request, our raw data and study protocols can be made available. Please e-mail morgan.valley@colostate.edu.

## Supplementary Materials

The following supporting information can be downloaded at: https://www.mdpi.com/article/10.3390/ijerph23080982/s1, File S1: Study eligibility screening survey and interview guide.
