# Supplementary material for: Perceived Occupational Benefits and Hazards of Soil Health Practices Among Colorado Farmers and Ranchers: A Qualitative Study
Source: Int J Environ Res Public Health. 2026 Jul 29;23(8):982. doi: 10.3390/ijerph23080982 (PMC13512217; doi:10.3390/ijerph23080982)
Supplement: Supplementary file 1 [file ijerph-23-00982-s001.zip › ijerph-4398887-supplementary.pdf]

Article

Perceived Occupational Benefits and Hazards of Soil Health Practices Among Colorado Farmers and Ranchers: A Qualitative Study

File S1. Study eligibility screening survey and interview guide

Survey questions

1. What state(s) do you farm or ranch in?
2. How long have you been farming or ranching?
    a. Less than a year
    b. 1-4 years
    c. 5-9 years
    d. 10-14 years
    e. 15-19 years
    f. 20-29 years
    g. 30+ years
3. How long has your family been farming or ranching, if applicable?
    a. Not applicable
    b. Less than a year
    c. 1-4 years
    d. 5-9 years
    e. 10-14 years
    f. 15-19 years
    g. 20-29 years
    h. 30-39 years
    i. 40-49 years
    j. 50-99 years
    k. 100 years or more
4. How much acreage do you farm or ranch?
    a. 1-9 acres
    b. 10-49 acres
    c. 50-179 acres
    d. 180-499 acres
    e. 500-999 acres
    f. 1,000+ acres
5. Do you own or lease the land you farm or ranch?
    a. Lease
    b. Own
    c. Both own and lease
    d. Other, please specify:
6. What is produced on your farm? Please select all that apply.
    a. Grains & Feeds
    b. Livestock Products
    c. Fruits
    d. Vegetables
    e. Legumes

f. Tree Nuts
g. Forestry Products
h. Fiber Products
i. Fuel Products
j. Other, please specify:

7. Do you do any soil testing?
   a. No
   b. Yes
   c. If yes, please briefly describe the tests you use and how often you test:
8. What practices, if any, do you use to build soil health? Please select all that apply.
   a. No till
   b. Strip till
   c. Minimal till
   d. Integrate livestock
   e. Rotational grazing
   f. Companion cropping
   g. Cover crops
   h. Optimize chemical input
   i. Diverse crop rotations
   j. Leave plant residue
   k. Use mulch
   l. Plant buffer strips
   m. Other, please specify:
   n. None of the above
9. How important, if at all, do you feel it is to build the soil's health where you farm or ranch?
   a. Not at all important
   b. Not very important
   c. A little important
   d. Somewhat important
   e. Very important
10. What is your age?
   a. Younger than 18
   b. 18-29
   c. 30-39
   d. 40-49
   e. 50-59
   f. 60-69
   g. 70 or older
11. What are the best times and days to interview you if you're selected to participate?

Semi-structured interview guide questions

Regenerative Farming Transition Process

1. What led you to start focusing on soil health?
   a. What were your motivations?
2. What changes have you experienced since then that you attribute to soil health practices? [If not covered, ask follow-up questions about:]
   a. Self
   b. Family
   c. Farm
   d. Social Relationships

e. Community

3. Have you noticed any changes in the way you view your work since you started prioritizing soil health?
    a. If so, would you please describe them?
4. What other changes do you hope to experience but haven't just yet?
5. How have your soil health practices been received by family and friends?
6. Has it impacted your social relationships and community?

Farmer Health & Safety

7. What connections, if any, do you see between soil and human health?
8. How would you describe the linkages between soil and farmer health?
9. How about impacts of dust on the farm and farmer health?
10. In what ways, if any, have your efforts to build soil health affected your or your family's health or well-being?
11. How about for workers on your farm, if any?
12. Have you noticed any changes in your physical health since adopting soil health practices? If so, would you please describe them?
13. How do soil health practices impact your mental wellbeing or stress levels compared to conventional farming methods?
14. Are there any specific soil health practices that you feel have had the most significant impact on your health or safety? What are the biggest reasons for that?
15. Has the use of personal protective equipment changed because of your soil health practices?
16. Have you noticed any changes in air quality or dust levels since adopting soil health practices? How might this affect respiratory health?
17. Do you think there are any potential long-term health benefits or risks associated with regenerative farming practices? What are they?

Wrap Up

18. What does it mean to you to work on the land?
19. Would you describe your work as meaningful? Why or why not?
20. What would it take for farming to have the most benefit for the world's health?
21. What aspects of society need to shift for this to happen?
22. Thank you for sharing your insights and time with me. Is there anything else you'd like to add about soil health, farming practices, or health and safety that we haven't covered?
